# Guanidine-Containing Polyhydroxyl Macrolides: Chemistry, Biology, and Structure-Activity Relationship

**DOI:** 10.3390/molecules24213913

**Published:** 2019-10-30

**Authors:** Xiaoyuan Song, Ganjun Yuan, Peibo Li, Sheng Cao

**Affiliations:** 1 College of Bioscience and Bioengineering, Jiangxi Agricultural University, Nanchang 330045, China; 2 School of Life Sciences, Sun Yat-sen University, 135 Xingang Road, Guangzhou 510275, China; lipb73@126.com

**Keywords:** guanidine, macrolide, bioactivity, antimicrobial, antibacterial, antifungal, structural diversity, structure-activity relationship, toxicity, azalomycin F

## Abstract

Antimicrobial resistance has been seriously threatening human health, and discovering new antimicrobial agents from the natural resource is still an important pathway among various strategies to prevent resistance. Guanidine-containing polyhydroxyl macrolides, containing a polyhydroxyl lactone ring and a guanidyl side chain, can be produced by many actinomycetes and have been proved to possess many bioactivities, especially broad-spectrum antibacterial and antifungal activities. To explore the potential of these compounds to be developed into new antimicrobial agents, a review on their structural diversities, spectroscopic characterizations, bioactivities, acute toxicities, antimicrobial mechanisms, and the structure-activity relationship was first performed based on the summaries and analyses of related publications from 1959 to 2019. A total of 63 guanidine-containing polyhydroxyl macrolides were reported, including 46 prototype compounds isolated from 33 marine and terrestrial actinomycetes and 17 structural derivatives. Combining with their antimicrobial mechanisms, structure-activity relationship analyses indicated that the terminal guanidine group and lactone ring of these compounds are vital for their antibacterial and antifungal activities. Further, based on their bioactivities and toxicity analyses, the discovery of guanidyl side-chain targeting to lipoteichoic acid of *Staphylococcus aureus* indicated that these compounds have a great potency to be developed into antimicrobial and anti-inflammatory drugs.

## 1. Introduction

Antimicrobial resistance has become a serious threat to human health and economic development [1]. Many strategies involving the development of new antimicrobial agents [2], the revival of old antibiotics [3,4], and combination therapy had been putting forward to fight or delay resistance [5]. On the one hand, our group has been researching the practice and law of drug combinations to prevent antimicrobial resistance [5,6,7]; on the other, we have been trying our best to discover new antimicrobial agents. Guanidine-containing polyhydroxyl macrolides can be generally biosynthesized by many actinomycetes [8,9,10], and all these compounds contain a lactone ring and a guanidyl side chain. Azalomycin F, a complex including three main compounds, F_3a_, F_4a_, and F_5a_, isolated from the broth of *Streptomyces hygroscopicus* var. *azalomyceticus* [11,12,13,14], was the first one reported. The planar structures of these three compounds were established by Namikoshi, Iwasaki, and Chandra et al. from 1982 to 1995 [13,14,15,16,17], and revised by Yuan et al. in 2011 [18]. Contemporaneously, many other guanidine-containing polyhydroxyl macrolides [19,20,21,22,23], such as niphimycin, RS-22, guanidylfungins, amycins, and shurimycins, were isolated from actinomycetes, especially streptomycetes. Although the planar structures of these compounds have been elucidated, their stereochemistries remained undetermined except the *E*-configuration of double bonds. Until 2013, the relative configurations of azalomycins F_5a_, F_4a_, and F_3a_ as three representatives of these compounds, together with seven new analogs, were reported by Yuan et al. [24,25]. Moreover, antimicrobial mechanisms indicated that the cell membrane was the main action site of these compounds against bacteria and fungi and that they could change the plasma membrane permeability and lead to the leakage of cellular substances [26,27,28,29]. In 2019, Yuan et al. [30] reported that azalomycin F_5a_ could bind to the polar head of cell-membrane phospholipid and target to lipoteichoic acid (LTA) to kill methicillin-resistant *Staphylococcus aureus* (MRSA). Up to 2019, approximately 48 guanidine-containing polyhydroxyl macrolides have been isolated from 33 marine and terrestrial actinomycetes. Moreover, 15 structural derivatives were synthesized. These compounds have broad-spectrum antimicrobial activity (especially Gram-positive bacteria and fungi) [31,32,33], anti-trichomonas [34], cytotoxicity [31,32], and so on. The structures and antimicrobial activities of some of them were partly involved in two previous reviews [31,32], while the complete structural diversity and bioactivities, acute toxicities, antimicrobial mechanisms, and structure-activity relationship of these compounds were not yet reported. Along with the clarification of some antimicrobial mechanisms and structure-activity relationships, we here presented a review on the chemistry and biology of these compounds discovered from 1959 to 2019 for exploring their potential in drug development.

## 2. Structural Diversity

**Figure 1:**
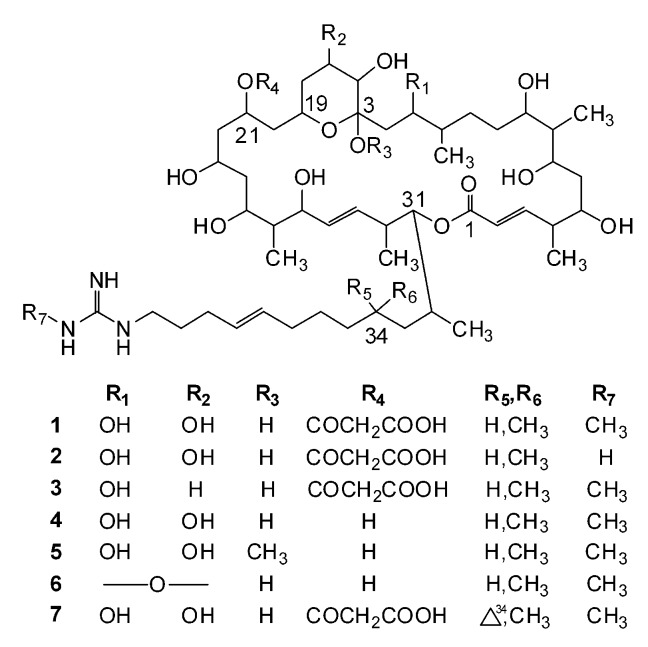
Chemical structures of 32-membered guanidine-containing polyhydroxyl macrolides (**1**–**7**).

**Figure 2:**
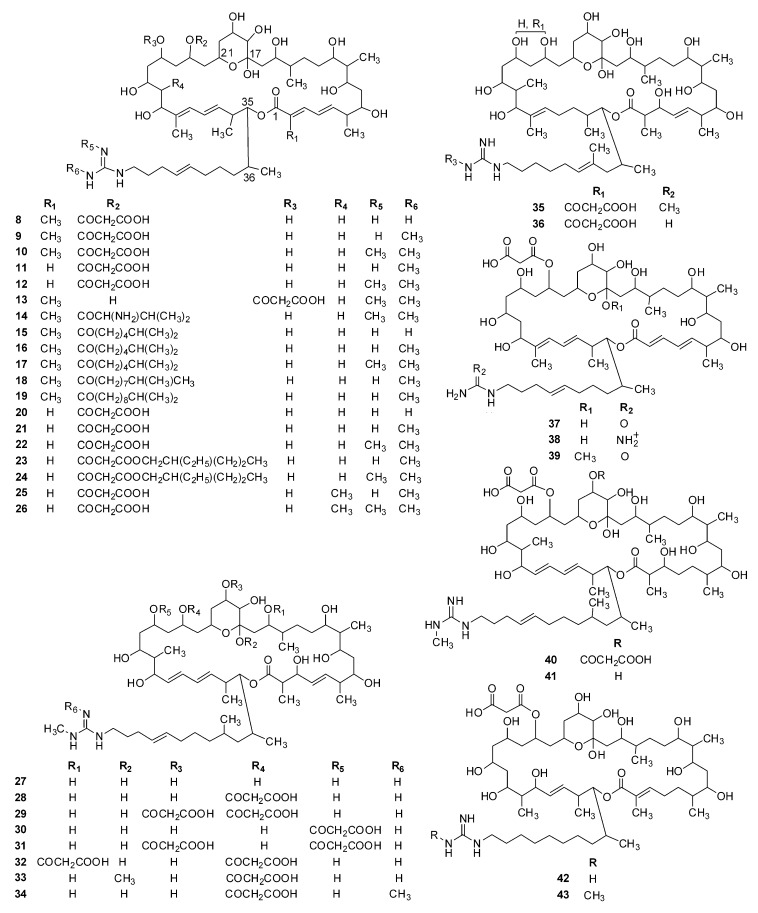
Chemical structures of 36-membered guanidine-containing polyhydroxyl macrolides (**8**–**43**).

**Figure 3:**
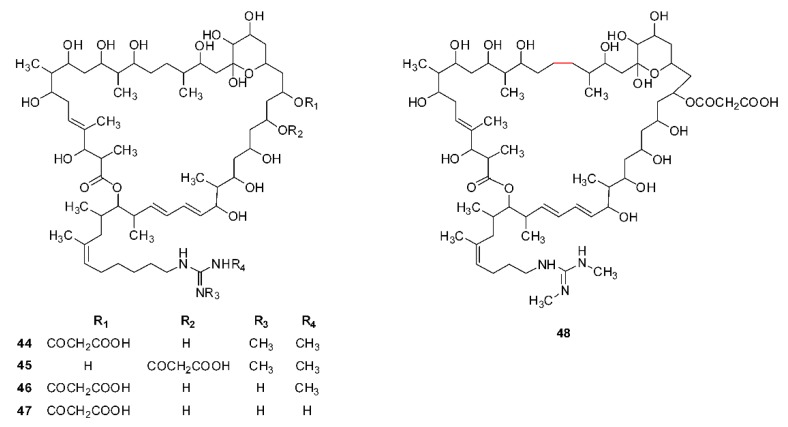
Chemical structures of 40-membered guanidine-containing polyhydroxyl macrolides (**44**–**48**).

Guanidine-containing polyhydroxyl macrolides are widely produced by actinomycetes, especially streptomycetes. Depending on the atom number composed of the lactone ring, they can be classified into three types: 32-, 36- and 40-membered polyhydroxyl macrolides. Until 2019, 48 guanidine-containing polyhydroxyl macrolides have been isolated and identified, which include seven 32-membered (**1**–**7**, Figure 1), thirty-six 36-membered (**8**–**43**, Figure 2), and five 40-membered (**44**–**48**) macrolides (Figure 3). All these compounds have a guanidyl side chain and a lactone ring, which includes a six-membered hemiketal ring, while there are differences at (1) the chain length (nine or eleven carbons), methyl number (one or two), and double bond position of guanidyl side chains [35]; (2) the atom number composed of the lactone ring (32, 36, or 40); (3) the number of malonyl monoesters (1 or 2), and their sites (C-19, C-23, or C-25) linking at the lactone ring; (4) the hydroxyl, methyl, and double bond numbers of the lactone ring. Simultaneously, all hydroxyl and methyl groups, except for those on six-membered hemiketal ring, present 1,3-, 1,5-, 1,7-, or 1,9- substitution characteristics, which include 1,3,5-, 1,5,7-, 1,3,5,7-, and 1,3,5,7,9- substitutions, and so on, and double bonds or carbonyl groups can be considered as a potential hydroxyl group as they can be, respectively, formed by the dehydration or oxidation of hydroxyl groups. These structural characterizations can be interpreted by the biosynthesis of guanidine-containing macrocyclic polyketides [8,9,10] and are very helpful for the structural elucidation of these compounds. However, the natural existence of compounds **37** and **39** cannot be explained as they share the same origin for guanidinobutanoate starter units in their biosynthesis pathways [9].

Moreover, compound RP 63834 (**48**, Figure 3) has a 41-membered lactone ring and a guanidyl side-chain containing eight carbons [36], which is different from the general character (a 32-, 36-, or 40-membered ring and a 9- or 11-carbon guanidyl side-chain) of other polyhydroxyl macrolides and is not in accordance with the rule that the positions of the ketone group are (n−2)/2 position in n-membered macrocyclic lactones [35]. Maybe, one of three methylene at C_46_, C_48_, and C_49_ position [36] should be assigned to its guanidyl side-chain, and compound RP 63834 is a 40-membered polyhydroxyl macrolide. Thereby, we deduced that compound RP 63834 was likely a compound **44**. The numbers, names, and corresponding sources and references of all these compounds are shown in Table 1.

**Figure 4:**
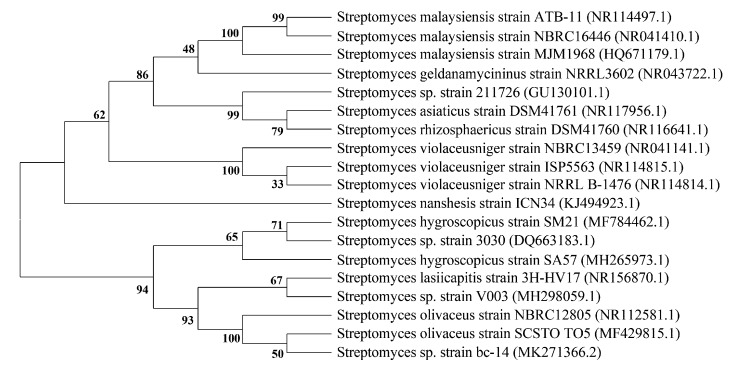
Neighbor-joining tree based on the 16*S* rRNA gene sequences of some strains producing guanidine-containing polyhydroxyl macrolide. Some similar strains belonging to the same species of these strains, which have no 16*S* rRNA gene sequences, were used.

Among 33 guanidine-containing polyhydroxyl macrolide-producing strains (Table 1), sixteen belong to *Streptomyces hygroscopicus*, ten are unidentified species of streptomycete genus, and other strains are *Streptomyces lasiicapitis* 3H-HV17(2)T [37], *Streptomyces malaysiensis* MJM1968 [27], *Streptomyces olivaceus* Tü 4018 [38], *Streptomyces violaceoniger* TÜ 905 [39], *Streptomyces violaceusniger* RS-22 [40], and two actinomycete strains HIL Y-9120362 and MT2617-2 [41,42]. To understand the affinity of these strains, the phylogenetic tree (Figure 4) was constructed using the neighbor-joining algorithms (some similar strains belonging to the same species of these strains, which have no 16*S* rRNA gene sequences, were used) [43]. Briefly, their 16*S* rRNA gene sequences were aligned against sequences of reference strains using the BLAST program (http://www.ncbi.nlm.nih.gov/). All the selected DNA multiple sequences were matched by means of software package Clustal_X 1.83 [44], and evolution distances were calculated using the Kimura2-Parameter model of MEGA version 6.0 [45]. Based on 1000 replicates, the confidence coefficient of the phylogenetic tree was evaluated using bootstrap analysis [46]. From Figure 4, there are no obvious distribution rules of species and genera between these guanidine-containing polyhydroxyl macrolides and their producing strains. Namely, the same macrolide can be produced by several species and genera with different genetic distances.

These molecules contain many chair centers (more than eighteen). As they have lager flexibility attributed to the larger ring and the longer side-chain, their single crystals were hardly obtained for determining the stereochemistry using an x-ray single diffraction method [15]. Simultaneously, other methods [68], such as optical rotatory dispersion (ORD), vibrational circular dichroism (VCD), and electrostatic circular dichroism (ECD), were also difficult to have assigned their absolute configurations because of the complexity and flexibility of these compounds. Thereby, most compounds only presented their planar structures except for the relative configurations of azalomycin F analogs and derivatives (**8**–**10**, **13**–**19**) [24,25] and the proposed absolute configurations of niphimycin analogs (**27**–**33**) [53].

**Figure 5:**
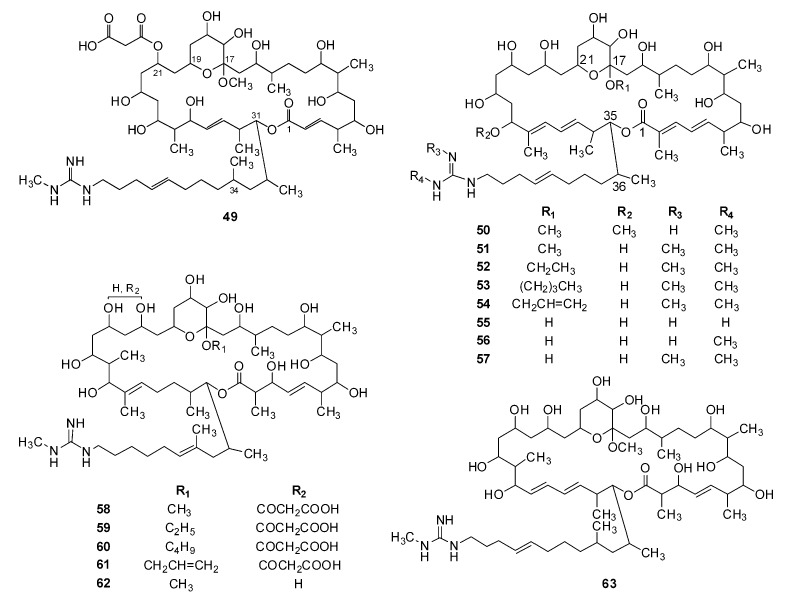
Structural derivatives (**49**–**63**) of some guanidine-containing polyhydroxyl macrolides.

Moreover, 15 derivatives (**49**–**63**, Figure 5) were synthesized from copiamycin, azalomycin F, guanidylfungin A, and niphimycin, which mainly involved the etherification of hemiketal hydroxyl and/or the hydrolysis of malonyl moiety. Their numbers, names, and corresponding raw materials and references are listed in Table 2.

## 3. Spectroscopic Characterization

As the chemical structures of these compounds have many similar fragments and groups, many NMR signals are close to each other, and some even overlap. These will increase the difficulties of their structural elucidations, while some regularity spectroscopic characterizations could be summarized, which would be very conducive to the structural elucidations of these compounds.

For the guanidyl side-chain, their guanidyl carbon signals at 157.3 to 158.3 ppm are easily observed from their ^13^C NMR spectra, and their chemical shifts decrease from approximately 158.7, 158.3 to 157.3 when the methyl number linking with guanidino nitrogen increases from 0, 1 to 2 [18,22,25,53]. These are also confirmed by the proton signals of N-methyl at 2.8 to 2.9 ppm on the ^1^H NMR spectra. Moreover, the stereochemistry of chain double bond is generally oriented in *E*-configuration. This is hardly established from the NMR spectrometric data due to overlapping signals; however, it can be confirmed by comparing the band at 969 cm^−1^ in the IR spectrum with the spectral data in the book [18,72].

For the lactone ring, many methyl and oxygenated methine signals can be observed from their ^13^C NMR spectra. As all these compounds share polyketide biosynthesis pathway [8,9,10,53], some general substitution characteristics of the hydroxyls and methyl groups linking on the lactone ring and guanidyl side-chain, such as 1,3-, 1,5-, 1,7-, 1,9-, 1,3,5-, 1,3,5,7-, 1,5,7-, and 1,3,5,7,9- substitutions, are very useful for their structural elucidations and NMR signal assignments. Some NMR data, including a quaternary hemiketal carbon at 99 to 100 ppm, the carbon at about 80 ppm and proton at 4.81 ppm of oxygenated methine forming lactone, and the proton at 5.23 ppm of oxygenated methine linking malonyl moiety will be also helpful for their structural elucidations. Furthermore, the presence or not of a conjugated diene and/or an *α*, *β*, *γ*, and *δ*-unsaturated acid (or ester) group can be easily deduced from whether there are UV absorption maxima at 240 nm (lg*ε* more than 4) and/or 269 nm (lg*ε* more than 4) [13,18]. For the malonyl moiety, the carbon signal of methylene is hardly observed (sometimes a little) in a protic solvent, such as methanol-*d*_4_, as the keto-enol tautomerization rapidly occurs [18,53], while it is easily detected in an aprotic solvent, such as DMSO-*d*_6_. Simultaneously, the two protons present multiple peaks in the ^1^H NMR spectrum as they quickly exchange with deuterium at the measurement conditions, especially at higher temperatures [18].

## 4. Bioactivity

Guanidine-containing polyhydroxyl macrolides have been experimentally documented to possess broad-spectrum antibacterial and antifungal activities, and they can remarkably inhibit the growth of Gram-positive bacteria, yeast, and fungi (Table 3) [25,28,34,38,51,53,58,64]. Moreover, they also show antitrichomonal and antitumor activities [25,73,74].

Azalomycin F has remarkable bioactivities against Gram-positive bacteria, yeast, fungi, and protozoa [17,34,73,75], and some clinical studies on its anti-trichomoniasis and anti-candida infectious effects were performed [34], while no corresponding drug was approved in the clinic. Stefanelli et al. discovered that niphimycin (**28**) and azalomycin F (**8**–**10**) could inhibit the type-I interleukin-1 receptor [55]. Moreover, they had remarkable activities against phytopathogenic fungi, such as *Fusarium oxysporum*, *Fusarium moliniforme*, *Asparagus officinalis*, *Rhizoctonia solani, Colletotrichum gloeosporioides*, and *Alternaria mali* [17,36].

Hamagishi et al. [48] discovered that copiamycin A (**1**), azalomycin F (**8**–**10**), and scopafungin (niphimycin, **28**) could inhibit the secretion of gastric acid in the gastric parietal cells of rats by inhibiting H^+^/K^+^-ATPase with the IC_50_s of 15.7, 16.4, and 35.9 μg/mL, respectively. The inhibitory potency of copiamycin was found to be comparable to that of omeprazole and SCH-28080, both specific inhibitors of the gastric H^+^/K^+^-ATPase in vitro and in vivo [48].

In addition, Reusser [56] proposed that niphimycin (scopafungin, **28**) was an inhibitor of mitochondrial oxidative phosphorylation and respiration, and mainly a decoupling agent for oxidative phosphorylation. Furthermore, Mogi et al. [81] discovered that niphimycin had inhibitory activity against NADH dehydrogenase (NDH-II), and deduced that niphimycin had a great potential to become an antibacterial drug as it showed no severe effect on mammalian respiratory enzymes.

Using an NIH3T3 cell line, a screening system for Ras signal inhibitors was developed to search for anti-cancer agents by Futamura et al. [74]. Malolactomycin D (**47**) was identified as a selective inhibitor of Ras-responsive transcription. The expression of matrix metalloproteinases MMP-1 and MMP-9 in NIH3T3 cells line could be reduced by treatment with malolactomycin D at the translational and transcriptional levels, and this was achieved likely by inhibiting the activation of p38 mitogen-activated protein kinase (MAPK) and c-Jun N-terminal kinase (JNK) [82,83]. As MMPs contribute to tumor growth, invasion, and metastasis by promoting the degradation of extracellular matrix and maintaining the tumor microenvironment [84,85], malolactomycin D suppressing the transformation activity of Ras-transformed cells by inhibiting the expression of Ras-inducible genes, such as MMP-1 and MMP-9, indicated that it was expected to be a new anticancer agent with high efficiency and low toxicity.

Ko et al. [40] isolated a phospholipase C inhibitor (PLC) from the culture medium of actinomycete MT2617-2 and named it as MT2617-2B, which produced its two isomers having the same molecular weight by standing in methanol solution at room temperature, copiamycin and niphithricin A. Besides antimicrobial activities against *S. aureus* and *C. albicans*, MT2617-2B had a remarkable inhibitory activity against phospholipase C with the IC_50_ values against PLC-*γ*1 and PLC-*β*1 of 25 and 50 μg/mL, respectively.

## 5. Acute Toxicity

To understand the safety of these compounds, the median lethal dose (LD_50_) and maximal tolerable dose (MTD or LD_0_) of some guanidine-containing polyhydroxyl macrolides were determined. As shown in Table 4, the LD_50_ or LD_0_ doses of each compound successively decreased from oral, subcutaneous, intraperitoneal to intravenous administrations. Moreover, Benziger and Edelson reported that azalomycin F administered intravaginally presented limited absorption [86]. Thereby, we deduced that azalomycin F administered orally was likely difficult to be absorbed, and this might be in accordance with the experimental results of its acute toxicities in different administrations. It was inexplicable that the LD_50_ or LD_0_ of neocopiamycins A and B administered intraperitoneally were more than 1000 mg/kg, which indicated that they had low toxicity, while the LD_50_ or LD_0_ of neocopiamycins A and B administered intravenously were only more than 30 or 25 mg/kg. Moreover, these compounds, except for neocopiamycins A and B in Table 4, had similar acute toxicities when they were administrated intraperitoneally. This indicated that their toxicities might be attributed to the lactone ring and guanidyl side-chain, which were also mainly responsible for their antimicrobial activities, and likely had nothing to do with the size of lactone ring and the numbers of hydroxyl and methyl groups. It was worth noting that the purities of compounds or mixtures used for the acute toxicity test would fluctuate the experimental results; however, very few publications have provided this information.

## 6. Antimicrobial Mechanisms

As these compounds had remarkable inhibitory activities against Gram-positive bacteria and fungi, related researches mainly focused on antibacterial and antifungal mechanisms. Previous works indicated that cell membrane was the main action site of them against bacteria and fungi, and these compounds could change the plasma membrane permeability and lead to the leakage of cellular substances [26,27,28,30].

### 6.1. Antibacterial Mechanisms

As Sugawara reported [75], azalomycin F could lead to the leakage of cellular substances to kill *Bacillus subtilis*, while detailed mechanisms had not been further reported because their chemical structures were not clear at that time. Inspired by the fact that the antimicrobial activities of azalomycin F and copiamycin could be reversed in the same manner by the phospholipid fraction of the bacteria, and various phospholipids, such as phosphatidylglycerol (PG) and phosphatidylcholine [89], Yuan et al. [26] discovered that azalomycin F_5a_, the main component of azalomycin F, could lead to the leakage of cellular substances possibly by increasing permeability to kill *S. aureus* and confirmed that cell-membrane lipids, especially 1,2-dihexadecanoyl-*sn*-glycero-3-phospho-(1′-*rac*-glycerol) (DPPG), might be important targets of azalomycin F_5a_ against *S. aureus* after its relative configurations were assigned [24,26]. Further researches indicated that azalomycin F_5a_, increasing the cell membrane permeability of *S. aureus*, was likely achieved by the synergy of its lactone ring binding to the polar head of phospholipid and its guanidyl side-chain targeting to lipoteichoic acid (LTA), and which had eventually led to the autolysis of *S. aureus* cells [30]. The compositional analysis indicated that PG, lysyl-phosphatidylglycerol (LPG), and cardiolipin (CL) were three major components of *S. aureus* cell-membrane phospholipid, and PG was the largest one [90,91]. Simultaneously, the content of lysyl-DPPG in the cell-membrane lipids would increase when *S. aureus* was resistant to daptomycin [90]. Thereby, molecular dynamics simulation, showing that azalomycin F_5a_ had greater adhesive force to plasma membrane assembled by DPPG plus lysyl-DPPG than by DPPG, indicated that azalomycin F_5a_ likely had greatly antagonistic activity to daptomycin-resistant *S. aureus* strains, and then proposed that these compounds had a great potency to be developed into new antimicrobial agents as LTA is also an important target for new antibiotics [92,93].

### 6.2. Antifungal Mechanism

Although these compounds can change the cell membrane permeability of microbe and lead to the leakage of cellular substances, there are different mechanisms of them against bacteria and fungi as the components of their cell envelopes are different.

Sugawara [94] discovered that azalomycin F could cause the leakage of cellular substance from the cells of *C. albicans* and the lysis of rabbit erythrocytes, and strongly inhibit amino acid incorporation into cellular protein and oxidative deamination of amino acid metabolism, but not decarboxylation and transamination. Simultaneously, it insignificantly inhibited the incorporation of phosphate into nucleic acids and the glycolytic pathway and did not exert any noticeable inhibition in cell-free protein-synthesizing systems of *E. coli*, rat liver, and *C. albicans*, and mitochondrial enzyme systems. Thereby, the cell surface was proposed as the primary site of azalomycin F acting on *C. albicans* [94]. Moreover, antifungal mechanisms of other guanidine-containing polyhydroxyl macrolides also confirmed that these compounds, such as niphimycins and copiamycins, could act on the cell membrane of fungi and alter their membrane permeability to cause the leakage of cellular components [27,28,89]. Further researches proposed that copiamycin and zalomyci F disrupted the cell membrane of fungi by binding to the cell-membrane phospholipids [89]. Thorough antifungal mechanism indicated that a synergistic combination of direct plasma membrane damage and oxidative stress was a cause of antifungal activity of niphimycin against *Saccharomyces cerevisiae* [29], and proposed that niphimycin disrupted the plasma membrane by directly interacting with phospholipids, such as phosphatidylcholine, but did not interact with ergosterol, a molecular target of amphotericin B. At the same time, Nakayama et al. [29], depending on the differences in the structures of niphimycin and amphotericin, suggested that the ability of niphimycin damaging the plasma membrane and/or generating ROS residues was primarily attributed to the alkyl side chain and terminal guanidine. In addition, Uno et al. [77] inferred that copiamycin, a 32-membered guanidyl polyol macrolide, had ionophoretic activity and could form a conformation with a ring or cavity that focuses the oxygens in lactone ring with various cations into a complex.

## 7. Antimicrobial Structure-Activity Relationship

As antimicrobial activity is one of the most important bioactivities of these compounds, most of them presented their minimum inhibitory concentration (MIC) against bacteria and fungi (Table 3) when they were discovered. Thereby, the structure-activity relationships of these compounds against bacteria and fungi can be summarized as follows:

(1) The atom number composed of the lactone ring is less important for their antimicrobial activities [50,66,86] and acute toxicity (Table 4). It is worth noting that 32-membered guanidine-containing polyhydroxyl macrolide TCM-34 shows remarkable antifungal activity (MIC, 1.6 to 3.1 μg/mL), while presents very weak antibacterial activity (MIC, more than 100 μg/mL) [52].

(2) Antimicrobial activity is significantly affected by the guanidyl side-chain, especially by the terminal guanidine group, which is a key for their antibacterial and antifungal activities. The substitution of guanidino residue to urea will lead to the loss of antibacterial activity and significantly narrow the antifungal spectrum [29,38,95], while the number of methyl groups linking on guanidine has a little or no effect on the antimicrobial activity [12,18,20]. Moreover, enough length (9 or 11 carbons) of the side chain is necessary for the antimicrobial bioactivity [96].

(3) The hydrolysis of the lactone ring will lead to the loss of antimicrobial activity [95]. The six-membered hemiketal ring plays an essential role in the antimicrobial activity, and the opening of a six-membered hemiacetal ring will remarkably decrease the antimicrobial activity [50]. Simultaneously, the etherification of C_17_ hydroxyl will slightly reduce the antimicrobial activity, and sometimes this decrease can be counteracted by the increase of antimicrobial activity due to the removal of the malonyl group [21,50,58,70,71].

(4) There is no significant influence on the antimicrobial activity when hydrogenation, methyl removal, or/and methyl substitution occur to the double bond of the lactone ring. Similarly, methyl substitution of the double bond on the guanidyl side-chain is less important for the antimicrobial activity [20,23,38,63].

(5) The introduction of malonyl moiety will reduce the antimicrobial activity [21,50,53,70,71,97]. The more the number of malonyl substitution, the weaker the antibacterial activity of these compounds. However, the position of malonyl substitution shows no influence on their antibacterial activities [53].

**Figure 6:**
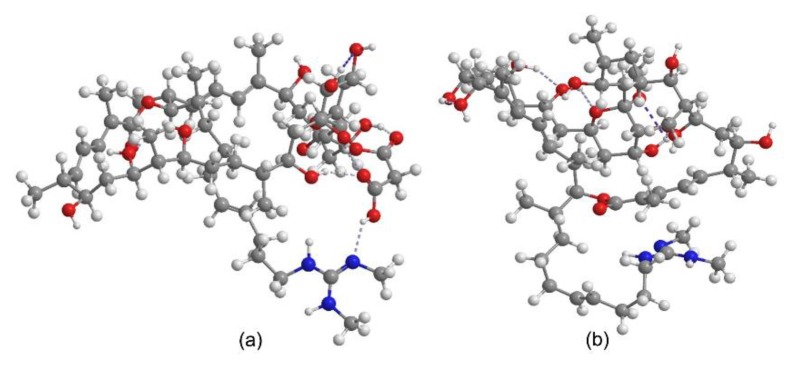
The 3D molecular structures of azalomycin F_5a_ (**a**) and 23-demalonyl azalomycin F_5a_ (**b**) obtained by ChemBio3D Ultra 12.0. (**a**) An intramolecular hydrogen bond or ionic bond (dotted line) is formed between the guanidyl (nitrogen atoms colored blue) of side-chain and the carboxyl group (oxygen atoms colored red) of malonyl monoester, but there is no bond formation in case of (**b**).

**Figure 7:**
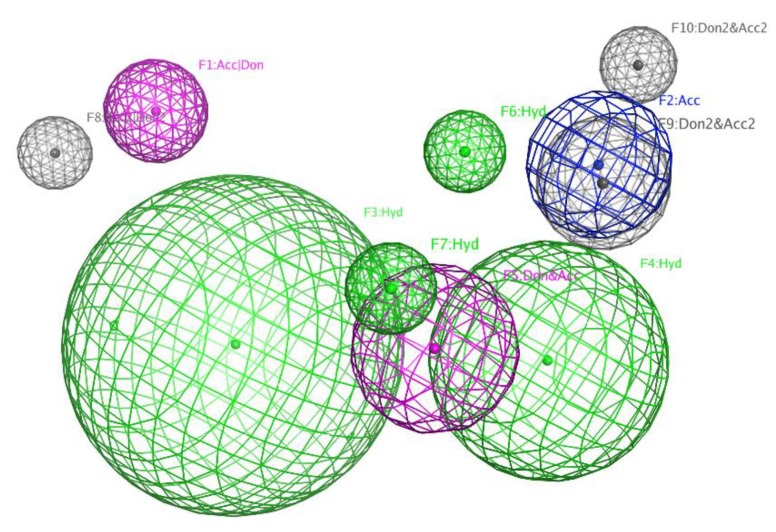
Pharmacophore model of 36-membered guanidine-containing polyhydroxyl macrolides obtained by Discovery Studio 3.5. Ten pharmacophore features were constructed, and was successively F1:Acc|Don (Hydrogen bond acceptor or donor); F2:Acc (Hydrogen bond acceptor); F3, F4, F6, and F7:Hyd (Hydrophobic region); F5:Don&Acc (Hydrogen bond donor and acceptor); F8 Acc2|Don2 (Hydrogen bond acceptor or donor projected); and F9 and F10:Don2&Acc2 (Hydrogen bond donor and acceptor projected).

As we reported [30], azalomycin F_5a_ could increase the cell membrane permeability of *S. aureus* and eventually lead to the autolysis of *S. aureus* cells, by the synergy of its lactone ring binding to the polar head of phospholipid and its guanidyl side-chain targeting to LTA, which is a vital anion component anchoring on the phospholipid bilayer of Gram-positive bacteria. This was in accordance with the above structure-activity relationship that the lactone ring and the terminal guanidyl side-chain were vital for the antimicrobial activity. As the carboxyl group of malonyl monoester can theoretically form an intramolecular hydrogen bond or ionic bond with the guanidyl of side-chain, the existence of malonyl will likely block the interaction between the guanidyl of side-chain and LTA. This was confirmed by their 3D molecular structures obtained by ChemBio3D Ultra 12.0 run with MM2 calculation (Figure 6) and by pharmacophore model of 36-membered guanidine-containing polyhydroxyl macrolides using Discovery Studio 3.5 (Figure 7), and could explain why the antimicrobial activity of azalomycin F was greatly reduced by phospholipids containing an acidic phosphoryl group [98]. Further, this can also explain why the introduction of malonyl will greatly reduce the antimicrobial activity and coincides with the above structure-activity relationship (5). From Figure 5, we can deduce that the substituted position (C-19, C-23, or C-25) of malonyl coincides with the spatial distance of the intramolecular salt or hydrogen bond formation between the terminal guanidine and the carboxyl group of malonyl monoester. This will likely reduce the interaction between the guanidyl of side-chain and LTA, and then reduce the antimicrobial activity of these compounds. Inspired by a previous publication [99], all these above indicate that the introduction of malonyl may be self-protection of actinomycetes producing guanidine-containing polyhydroxyl macrolide, through which they can be free from the poison and injury of secondary metabolites produced by themselves. Moreover, the demalonylation of these compounds not only increases the antimicrobial activity but also yields a basic compound, which has a better water solubility, especially for its hydrochloride [50,71].

## 8. Conclusions

To date, a total of 63 guanidine-containing polyhydroxyl macrolides were reported, including 48 prototype compounds isolated from 33 actinomycete strains and 15 structural derivatives. These compounds have various bioactivities, such as broad-spectrum antimicrobial activity, anti-trichomonas, anti-tumor, and inhibitory activities against H^+^/K^+^-ATPase, mitochondrial oxidative phosphorylation, NADH dehydrogenase, and phospholipase C, while they also have a little toxicity. Structure-activity relationships indicate that both the terminal guanidine group and the lactone ring are the key for their antimicrobial activities. As LTA anchoring to the cell membrane is an important polymer for the resistance to cationic antibiotics, the autolysin regulation, and the cell division of Gram-positive bacteria, LTA synthase gradually becomes a proposed drug target for the development of antibiotics against drug-resistant Gram-positive bacteria [92,93,100,101]. Thereby, the discovery of guanidyl side-chain targeting to lipoteichoic acid indicates these compounds have a great potency to be developed into antimicrobial and anti-inflammatory drugs.

## Figures and Tables

**Table 1:** Guanidine-containing polyhydroxyl macrolides from a natural source.

Compounds	Name	Sources	References (Publication Time)
**1**	Copiamycin A	*Streptomyces hygroscopicus* var. *crystallogenes* ATCC 19040	[47] (1965)
		*Streptomyces violaceoniger* TÜ 905	[39] (1981)
		*Streptomyces hygroscopicus* sp. M931-1	[48] (1991)
		*Actinomycete* MT2617-2	[40] (1996)
		*Streptomyces hygroscopicus* var. *crystallogenes* IFM 1136	[49] (1999)
**2**	Neocopiamycin A	*Streptomyces hygroscopicus* var. *crystallogenes*	[50] (1984)
		*Streptomyces hygroscopicus* var. *crystallogenes* IFM 1136	[49] (1999)
**3**	Neocopiamycin B	*Streptomyces hygroscopicus* var. *crystallogenes* IFM 1136	[49] (1999)
**4**	Demalonylcopiamycin	*Streptomyces hygroscopicus* var. *crystallogenes* IFM 1136	[49] (1999)
**5**	Demalonylmethylcopiamycin	*Streptomyces hygroscopicus* var. *crystallogenes* IFM 1136	[49] (1999)
**6**	Guanidolide A	*Streptomyces hygroscopicus* var. *crystallogenes* IFM 1136 *Streptomyces*	[49] (1999)
		*hygroscopicus* var. *crystallogenes*	[51] (1988)
**7**	TMC-34	*Streptomyces*. A-3030	[52] (1995)
**8**	Azalomycin F_3a_	*Streptomyces hygroscopicus* var. *azalomyceticus*	[11] (1960)
		*Streptomyces hygroscopicus* MSU/MN-4-75B	[17] (1995)
		*Streptomyces malaysiensis* MJM1968	[36] (2010)
		*Streptomyces* sp. 211726	[18] (2011)
**9**	Azalomycin F_4a_	*Streptomyces hygroscopicus* var. *azalomyceticus*,	[11] (1960)
		*Streptomyces hygroscopicus* MSU/MN-4-75B	[17] (1995)
		*Streptomyces malaysiensis* MJM1968,	[36] (2010)
		*Streptomyces* sp. 211726	[18] (2011)
**10**	Azalomycin F_5a_	*Streptomyces hygroscopicus* var. *azalomyceticus*,	[11] (1960)
		*Streptomyces hygroscopicus* MSU/MN-4-75B	[17] (1995)
		*Streptomyces malaysiensis* MJM1968,	[36] (2010)
		*Streptomyces* sp. 211726	[18] (2011)
**11**	2-Demethyl azalomycin F_4a_	*Actinomycete* sp. HIL Y-9120362	[39] (1995)
**12**	2-Demethyl azalomycin F_5a_	*Actinomycete* sp. HIL Y-9120362	[39] (1995)
**13**	25-Malonyl demalonyl azalomycin F_5a_ monoester	*Streptomyces* sp. 211726,	[25] (2013)
**14**	23-Valine demalonyl azalomycin F_5a_ ester	*Streptomyces* sp. 211726,	[25] (2013)
**15**	23-(6-Methyl) heptanoic acid demalonylazalomycin F_3a_ ester	*Streptomyces* sp. 211726,	[25] (2013)
**16**	23-(6-Methyl) heptanoic acid demalonylazalomycin F_4a_ ester	*Streptomyces* sp. 211726,	[25] (2013)
**17**	23-(6-Methyl) heptanoic acid demalonylazalomycin F_5a_ ester	*Streptomyces* sp. 211726,	[25] (2013)
**18**	23-(9-Methyl) decanoic acid demalonylazalomycin F_4a_ ester	*Streptomyces* sp. 211726,	[25] (2013)
**19**	23-(10-Methyl) undecanoic acid demalonylazalomycin F_4a_ ester	*Streptomyces* sp. 211726,	[25] (2013)
**20**	RS-22A	*Streptomyces violaceusniger* RS-22	[22,38] (1995)
**21**	RS-22B	*Streptomyces violaceusniger* RS-22	[22,38] (1995)
**22**	RS-22C	*Streptomyces violaceusniger* RS-22	[22,38] (1995)
**23**	Azalomycin F_4a_ 2-ethylpentyl ester	*Streptomyces* sp.211726	[18] (2011)
**24**	Azalomycin F_5a_ 2-ethylpentyl ester	*Streptomyces* sp.211726	[18] (2011)
**25**	Shurimycin A	*Streptomyces hygroscopicus* A1491	[23] (1992)
**26**	Shurimycin B	*Streptomyces hygroscopicus* A1491	[23] (1992)
**27**	Amycin B	*Streptomyces* sp. DSM 3816,	[21] (1990)
		*Streptomyces* sp. IMB7-145	[53] (2018)
**28**	Niphimycin (scopafungin)	*Streptomyces hygroscopicus* B-255	[54] (1967)
		*Streptomyces* sp. DSM 3816,	[21] (1990)
		*Streptomyces* sp.KP6107,	[28] (2013)
		*Streptomyces* sp. GE48009	[55] (1997)
		*Streptomyces hygroscopicus* var. *enhygrus*	[56,57] (1972)
		*Streptomyces hygroscopicus* var. *enhygrus* NRRL 3664	[58] (1986)
		*Streptomyces hygroscopicus* var. *enhygrus* var. nova UC-2397	[59] (1971)
		*Streptomyces* sp. IMB7-145	[53] (2018)
**29**	Amycin A	*Streptomyces* sp. DSM 3816	[21] (1990) [53] (2018)
**30**	25-Malonyl Demalonylniphimycin	*Streptomyces* sp. IMB7-145	[53] (2018)
**31**	19,25-Malony Demalonylniphimycin	*Streptomyces* sp. IMB7-145	[53] (2018)
**32**	15-Malonyl Niphimycin	*Streptomyces* sp. IMB7-145	[53] (2018)
**33**	17-O-Methylniphimycin	*Streptomyces* sp. IMB7-145	[53] (2018)
**34**	N′-methyniphimycin	*Streptomyces* spec. 57-13	[60] (1998)
**35**	Guanidyfungina A	*Streptomyces hygroscopicus* No. 662	[20,61] (1984)
**36**	Guanidyfungina B	*Streptomyces hygroscopicus* No. 662	[20,61] (1984)
**37**	Kanchanamycin A	*Streptomyces* strain Tü 4018, *Streptomyces lasiicapitis* sp. nov.	[38,62] (1996) [35] (2017)
**38**	Kanchanamycin C	*Streptomyces* strain Tü 4018, *Streptomyces lasiicapitis* sp. nov.	[38,62] (1996) [35] (2017)
**39**	Kanchanamycin D	*Streptomyces* strain Tü 4018, *Streptomyces lasiicapitis* sp. nov.	[38,62] (1996) [35] (2017)
**40**	Malonyl-4,5-dihydroniphimycin	*Streptomyces hygroscopicus* 15	[63] (2007)
**41**	Dihydroniphimycin	*Streptomyces hygroscopicus* 15	[64] (2000)
**42**	Polaramycin A	*Streptomyces hygroscopicus* LP-93	[65] (1997)
**43**	Polaramycin B	*Streptomyces hygroscopicus* LP-93	[65] (1997)
**44**	Malolactomycin A	*Streptomyces* sp. 83-634	[45,66] (1993)
**45**	Malolactomycin B	*Streptomyces* sp. 83-634	[45] (1993)
**46**	Malolactomycin C	*Streptomyces* KP-3144	[67] (1997)
**47**	Malolactomycin D	*Streptomyces* KP-3144	[67] (1997)
**48**	RP 63834	*Streptomyces* strain n* S-13361	[36] (1991)

**Table 2:** Structural derivatives (**49**–**63**) of some guanidine-containing polyhydroxyl macrolides.

Compounds	Derivatives Name	Raw Materials	References (Publication Time)
**49**	17-Methyl copiamycin	Copiamycin	[69] (1985)
**50**	17,29-Dimethyl demalonylazalomycin F_4a_	Azalomycin F_4a_	[58] (1986)
**51**	17-Methyl demalonylazalomycin F_5a_	Azalomycin F_5a_	[70] (2014)
**52**	17-Ethyl demalonylazalomycin F_5a_	Azalomycin F_5a_	[70] (2014)
**53**	17-Butyl demalonylazalomycin F_5a_	Azalomycin F_5a_	[70] (2014)
**54**	17-Allyl demalonylazalomycin F_5a_	Azalomycin F_5a_	[70] (2014)
**55**	Demalonylazalomycin F_3a_	Azalomycin F_3a_	[71] (2016)
**56**	Demalonylazalomycin F_4a_	Azalomycin F_4a_	[71] (2016)
**57**	Demalonylazalomycin F_5a_	Azalomycin F_5a_	[71] (2016)
**58**	17-Methyl guanidylfungin A	Guanidylfungin A	[69] (1985)
**59**	17-Ethyl guanidylfungin A	Guanidylfungin A	[69] (1985)
**60**	17-Butyl guanidylfungin A	Guanidylfungin A	[69] (1985)
**61**	17-Allyl guanidylfungin A	Guanidylfungin A	[69] (1985)
**62**	17-Methyl demalonylguanidylfungin A	Guanidylfungin A	[69] (1985)
**63**	17-Methyl demalonylniphimycin	Niphimycin	[58] (1986)

**Table 3:** Antibacterial activity of guanidyl-polyol macrolide antibiotics.

Compounds	Minimum Inhibitory Concentrations to Various Pathogenic Microorganisms (μg/mL)	References
**1**	*Sarcina flava* IFM 2242 (25), *Sarcina flava* IFM 2243 (3.12), 3 isolates of *Sarcina hansenii* (12.5–25.0), 4 isolates of *Sarcina lutea* (3.12–12.5), *Sarcina subflava* IFM 2116 (3.12), 3 isolates of *Sarcina ureae* (1.56–6.25), 16 isolates of *Candida albicans* (0.78–100), 3 isolates of *Candida guilliermondii* (100), *Candida tropicalis* IFM 40018 (100), *Candida krusei* IFM 40019 (100), *Candida parapsilosis* IFM 40020 (100), *Candida stellatoidea* IFM 40021 (100), *Cryptococcus neoformans* IFM 40037 (1.56), *C. neoformans* IFM 40038 (1.56), *C. neoformans* IFM 40047 (6.25), *Staphylococcus aureus* FDA 209P (12.5), *Geotrichum candidum* IFM 40068 (1.56), *Epidermophyton floccosum* IFM 40747 (0.39), *Microsporum canis* (3.13), *Microsporum gypseum* IFM 40727 (6.25), *Mucor hiemalis* (3.13), *Mucor Racemosus* (3.13), *Trichophyton mentagrophytes* Kamiyama (3.13), *Trichophyton rubrum* IFM 40732 (3.13), *Sporothrix schenckill* IFM 40750 (hyphal phase) (50), *Fonsecaea pedrosoi* IFM 40756 (100), *Histoplasnma capsulatum* IFM 40752 (hyphal phase) (0.78), 4 clinical isolates of *Trichophyton glabrata* (0.78~3.13), 14 clinical isolates of *Trichophyton vaginalis* (12.5 to >100)	[39,50,51,69,76,77]
**2**	8 isolates of *C. albicans* (1.56–6.25), *C. guilliermondii* IFM 40017 (3.13), *C. tropicalis* IFM 40018 (3.13), *C. krusei* IFM 40019 (3.13), *C. parapsilosis* IFM 40020 (3.13), *C. tropicalis* (3.13), *C. stellatoidea* (IFM 40021) (3.13), *Candida utillis* IFM 40099 (12.5), *C. neoformans* IFM 40037 (<0.78), *C. neoformans* IFM 40038 (<0.78), *C. neoformans* IFM 40047 (1.56), *G. candidum* IFM 40068 (1.56), *Torulopsis glabrata* IFM 40065 (6.25), *Trichophyton cutaneum* IFM 40066 (6.25), *Saccharomyces cerevisiae* sake IFM 40025 (12.5), *S. schenckill* IFM 40751 (yeast phase) (3.13), *Aspergillus flavus* 23 (6.25), *Aspergillus fumigatus* 25 (25), *Aspergillus nidulans* 21 (6.25), *Aspergillus niger* 22 (6.25), *Aspergillus oryzae* IFM 40607 (6.25), *Aspergillus versicolor* 26 (12.5), *Penicillium expansum* IFM 40619 (3.13), *E. floccosum* IFM 40747 (0.78), *M. canis* (1.56), *M. gypseum* IFM 40727 (1.56), *T. mentagrophytes* IFM 40737 (1.56), *T. mentagrophytes* Kamiyama (1.56), *T. rubrum* IFM 40732 (0.39), *S. schenckill* IFM 40750 (hyphal phase) (6.25), *F. pedrosoi* IFM 40756 (3.13), *H. capsulatum* IFM 40752 (hyphal phase) (0.78)	[50]
**3**	*A. flavus* 23 (6.25), *A. fumigatus* 25 (12.5), *A. nidulans* 21 (6.25), *A. niger* 22 (6.25), *A. oryzae* IFM-40607 (6.25), *A. versicolor* 26 (6.25), *Penicillium glaucum* 3-1 (12.5), *E. floccosum* IFM 40747 (<0.78), *M. canis* (3.12), *M. gypseum* IFM 40727 (3.12), *T. mentagrophytes* IFM 40737 (1.56), *T. rubrum* IFM 40732 (1.56), *Sporothrix schenckii* IFM 40750 (6.25), *S. schenckii* IFM 40751 (yeast phase) (6.25), *F. pedrosoi* IFM 40756 (3.12), 8 isolates of *C. albicans* (0.78~12.5), *C. guilliermondii* IFM 40017 (6.25), *C. tropicalis* IFM 40018 (6.25), *C. krusei* IFM 40019 (6.25), *Candida prarsilosis* IFM 40020 (6.25), *C. stellatoidea* IFM 40021 (3.12), *C. neoformans* IFM 40037 (<0.78), *C. neoformans* IFM 40038 (<0.78), *C. neoformans* IFM 40047 (<0.78), *C. utillis* IFM 40099 (6.25), *T. glabrata* IFM 40065 (<0.78)	[49]
**4**	*S. aureus* FDA 209P (12.5), *Bacillus subtillis* NIHJ PCI 219 (12.5), *Cochiobolus miyabeanus* IFO 5277 (3.2), *Alternaria mali* IFO 8984 (12.5), *Botryotinia fuckeliana* IFO 5363 (1.6), *Colletorichum lagenarium* IFO 7513 (1.6), *Pellicularia filamentosa* sp. Sasakill IFO 6258 (1.6), *Pyricularia oryzae* IFO 5994 (6.25), *A. oryzae* IFO 5239 (25), *T. mentagrophytes* IFO 6202 (6.25), *T. glabrata* IFM 40065 (<0.78), *S. cerevisiae* IFO 0304 (1.6), *C. albicans* IFO 1594 (1.6), *C. krusei* IFM 40019 (6.25), *C. prarsilosis* IFM 40020 (6.25), *C. stellatoidea* IFM 40021 (3.12), *C. neoformans* IFM 40037 (<0.78), *C. neoformans* IFM 40038 (<0.78), *C. neoformans* IFM 40047 (<0.78), *C. utillis* IFM 40099 (6.25)	[51]
**5**	*S. aureus* FDA 209P (3.12), *S. cerevisiae* IAM 4020 (6.25), *C. albicans*, *A. nidulans* 21 (12.5), *P. expansum* IFM 40619 (6.25), *T. mentagrophytes* IFM 40734 (0.78), *M. gypseum* IFM 40727 (0.78), *E. floccosum* IFM 40747 (0.78)	[51,69]
**6**	*A. nidulans* 21 (50), *Paecilomyces expansum* (100), *T. mentagrophytes* IFM 40734 (12.5), *M. gypseum* IFM 40727 (25), *E. floccosum* IFM 40747 (12.5)	[51]
**7**	*C. albicans* ATCC 48130 (3.1), *C. neoformans* 145 A (1.6), *A. fumigatus* TUKUBA 48130 (3.1), *T. mentagrophytes* (3.1), *T. rubrum* (1.6)	[52]
**8**	Ten isolates of *S. aureus* (4–8), *B. subtilis* PCI 219 (12.5), *S. aureus* 209 P (6.25–12.5), *S. lutea* (6.25), *Corynebacterium xerosis* (6.25), *Mycobacterium smegmatis* ATCC607 (25), *C. albicans* Yu 1200 (1.56), *S. cerevisiae* (1.56–3.12), *Torula utilis* (1.56–3.12), *C. neoformans* (1.56), *Kloeckera africana* (1.56), *Trichophyton asteroids* (3.12), *Trichophyton interdigitale* (1.56), *A. oryzae* (6.25), *A. niger* (6.25~12.5), *Penicillium notatum* (1.56–3.12), *P. oryzae* (0.78–1.56), *Ophioborus miyabeanus* (0.78), *Alternaria kikuchiana* (1.56), *Sclerotinia libertiana* (0.78–1.56), *Fusarium lycopersici* (3.12), *Fusarium lini* (1.56~3.12), *Ceratostomella fimbriata* (1.56), *Trichomonas vaginalis* (12.5)	[12,78]
**9**	Ten isolates of *S. aureus* (4~12.5), *C. albicans* (12.5), *A. fumigatus* IAM 2046 (25), *A. fumigatus* IAM 2046 (MCC, >200)	[58,78]
**10**	Ten isolates of *S. aureus* ATCC 33592 (4~8)	[78]
**11**	The diameter of the zone of inhibition (mm): *C. albicans* (11), *Penicillium digitatum* (15), *Fusarium culmorum* 100 (17), *A. mali* P37 (20), *P. oryzae* K02 (16), *Leptosphaeria oryzae* J02 (23), *Pellicularia sasakill* J03 (28), *Pseudomonas herpotrichoides* 008 (19), *Neurospora crassa* SGF-18 (11), *Botrytis cinereal* E02 (22), *B. cinereal* A06 (13), *B. cinereal* D01 (17), *Phytophthora infestans* J08 (13)	[41]
**12**	The diameter of the zone of inhibition (mm): *C. albicans* (slight), *P. digitatum* (14), *F. culmorum* 100 (16), *A. mali* P37 (18), *P. oryzae* K02 (20), *L. oryzae* J02 (27), *P. sasakill* J03 (25), *P. herpotrichoides* 008 (16), *N. crassa* SGF-18 (12), *B. cinereal* E02 (20), *B. cinereal* A06 (12), *B. cinereal* (D01) (13), *P. infestans* J08 (15)	[41]
**13**	*C. albicans* ATCC 10231 (3.13), *S. aureus* S014 (0.39), *B. subtilis* S028 (0.20), *Esherichia coli* S002 (3.13)	[25]
**14**	*C. albicans* ATCC 10231 (6.25), *S. aureus* S014 (1.56), *B. subtilis* S028 (0.39), *E. coli* S002 (6.25)	[25]
**15**	*C. albicans* ATCC 10231 (3.13), *S. aureus* S014 (0.78), *B. subtilis* S028 (0.39), *E. coli* S002 (3.13)	[25]
**16**	*B. subtilis* S028 (0.20), *C. albicans* ATCC 10231 (1.56), *E. coli* S002 (6.25), *S. aureus* S014 (1.56)	[25]
**17**	*B. subtilis* S028 (0.78), *C. albicans* ATCC 10231 (1.56), *E. coli* S002 (12.5), *S. aureus* S014 (0.78)	[25]
**18**	*B. subtilis* S028 (0.39), *C. albicans* ATCC 10231 (3.13), *E. coli* S002 (25), *S. aureus* S014 (0.39)	[25]
**19**	*B. subtilis* S028 (0.39), *C. albicans* ATCC 10231 (3.13), *E. coli* S002 (3.13), *S. aureus* S014 (0.39)	[25]
**20**	*S. aureus* (6.25~12.5), *Streptococcus pyogenes* Cook (25), *C. neoformans* KC-201 (3.13), *C. albicans* KC-07 (3.13), *C. tropicalis* KC-104 (1.56), *C. prarsilosis* KC-110 (6.25), *C. glabrata* KC-308 (6.25), *A. flavus* KA-06 (12.5), *A. fumigatus* KA-01 (12.5), *T. mentagrophytes* KD-114 (12.5), *T. rubrum* KD-114 (12.5), *M. canis* KD-305 (12.5), *M. gypseum* KD-318 (12.5)	[40]
**21**	*S. aureus* (6.25~12.5), *S. pyogenes* Cook (12.5), *C. neoformans* KC-201 (3.13), *C. albicans* KC-07 (3.13), *C. tropicalis* KC-104 (3.13), *C. prarsilosis* KC-110 (6.25), *C. glabrata* KC-308) (6.25), *A. flavus* KA-06 (12.5), *A. fumigatus* KA-01 (12.5), *T. mentagrophytes* KD-114 (12.5), *T. rubrum* KD-114 (12.5), *M. canis* KD-305 (12.5), *M. gypseum* KD-318 (12.5)	[40]
**22**	*S. aureus* (6.25~12.5), *S. pyogenes* Cook (25), *C. neoformans* KC-201 (3.13), *C. albicans* KC-07 (6.25), *C. tropicalis* KC-104 (3.13), *C. prarsilosis* KC-110 (6.25), *C. glabrata* KC-308 (6.25), *A. flavus* KA-06 (12.5), *A. fumigatus* KA-01 (25), *T. mentagrophytes* KD-114 (12.5), *T. rubrum* KD-114 (12.5), *M. canis* KD-305 (12.5), *M. gypseum* KD-318 (12.5)	[40]
**23**	*C. albicans* ATCC 10231 (2.34)	[18]
**24**	*C. albicans* ATCC 10231 (12.5)	[32]
**25**	*B. subtillis* (3.1), *S. lutea* (3.1), *S. aureus* 209P (1.56), *Botrytis fragilis* (6.2), *C. neoformans* (1.56), *T. mentagrophytes* (3.1), *A. fumigatus* (3.1), *A. mali* (3.1), *Fusarium oxysporum* (12.5), *B. cinereal* (0.78), *P. oryzae* (0.78), *Rhizoctonia solani* (0.78), *C. albicans* (3.1)	[23]
**26**	*B. subtillis* (3.1), *S. lutea* (3.1), *S. aureus* 209P (1.56), *Bacteroides fragilis* (12.5), *C. neoformans* (1.56), *T. mentagrophytes* (3.1), *A. fumigatus* (6.2), *A. mali* (3.1), *F. oxysporum* (12.5), *B. cinereal* (0.78), *P. oryzae* (0.78), *R. solani* (1.56), *C. albicans* (6.2)	[23]
**27**	*T. mentagrophytes* (3.91), *T. rubrum* (3.91), *M. canis* (1.95), *C. albicans* (3.91), *A. niger* (1.95), 3 isolates of *S. aureus* (3.13), *S. pyogenes* 308 (6.25), *S. pyogenes* 77 A (6.25), *Staphylococcus. faecium* D (6.25)	[21,53,60]
**28**	Five isolates of *C. albicans* (1.56~12.5), *Trichophyton gypseum*, (1.56–15.6), *Fusarium graminarum* (3.12~7.8), *B. subtillis* (6.25). 15.6), *B. subtilis* (31.25)., *T. mentagrophytes* (3.91–10), *T. rubrum* (1.95), *T. asteroids* UC-4775 (1), *M. canis* (0.48), *M. canis* UC-1395 (10), *Cryptococcus immnzitis* UC-1119 (1), *C. neoformans* UC-1139 (1), *A. niger* (3.91), *A. fumigatus* IAM 2046 (12.5), *B. subtilis* ATCC 6633 (16), *B. subtilis* UC-564 (4), *B. dermatitidlis* UC-1911 (1), *F. culmorum* JP 15 (25), *Geotrichutim* sp. UC-1207 (1), *Hormodendrulmn compactum* UC-1222 (1), *Hormodendrulmn capsulatum* UC-1220 (0.1), *Nocardia asteroidles* UC-2052 (10), *Phialophora verrlucosai* UC-1807 (1), 9 isolates of *S. aureus* (6.25–16), *Streptococcus hemolyticus* UC- 15 (31), *S. pyogenes* 308 (25), *S. pyogenes* 77 A (25), *S. faecium* D (50), *Staphylococcus faecalis* UC-3235 (31), 3 isolates of *S. epidermidis* (32), *S. schenlckii* UC-1364 (10), 7 isolates of *Enterococcus faecalis* (32~64), *A. fumigatus* IAM 2046 (MCC, >200)	[8,21,53,59,60,63,79]
**29**	*T. mentagrophytes* (7.81), *T. rubrum* (7.81), *M. canis* (1.95), *C. albicans* (31.2), *A. niger* (7.81), 3 isolates of *S. aureus* (50), *S. pyogenes* 308 (100), *S. pyogenes* 77 A (100), *Penicillium chrysogenum* (7.81), *B. subtilis* (50), *Micrococcus luteus* (6.25),	[21,53,58,59,60,63,79]
**30**	Three isolates of *S. epidermidis* (16–64), 5 isolates of *S. aureus* (8~16), *E. faecalis* (64), *E. faecium* (64), *C. albicans* ATCC 10231 (16)	[53]
**31**	Four isolates of *S. aureus* (64), *C. albicans* ATCC 10231 (64)	[53]
**32**	Five isolates of *Staphylococcus epidermidis* (16~32), *S. epidermidis* 12-8 (64), *C. albicans* ATCC 10231 (16)	[53]
**33**	Five isolates of *S. epidermidis* (8–32), 2 isolates of *S. epidermidis* (16~32), *E. faecalis* ATCC 29212 (32), 2 isolates of *E. faecalis* (64), 3 isolates of *E. faecium* 12-3 (64), *C. albicans* ATCC 10231 (8)	[53]
**34**	The diameter of inhibition zone (mm): *C. albicans* (18), *F. culmorum* JP 15 (18), *Glomerella cingulate* (13), *Klyuveromyces marxianus* IMET 25148 (19), *P. notatum* JP 36 (15), *Sporobolomyces salmonicolor* SBUG 549 (24)	[60]
**35**	*S. aureus* FDA 209P (12.5), *C. albicans* IAM 4888 (25), *S. cerevisiae* IAM 4020 (50), *A. fumigatus* IAM 2153 (50), *T. mentagrophytes* (12.5), *Sporotrichum schenckii* (25), *Paecilomyces variotii* IAM 5001 (6.25), *C. albicans* (MCC, >200), *A. fumigatus* (MCC, >200)	[20]
**36**	*S. aureus* FDA 209P (50), *B. subtillis* PCI 219 (50), *C. albicans* IAM 4888 (100), *A. oryzae* (25), *A. fumigatus* (MCC, >200), *T. mentagrophytes* (6.25), *C. albicans* (MCC, >200)	[20]
**37**	*S. aureus* ATCC 11632 (30), *Pseudomonas fluorescens* ATCC 13525 (3), *Aspergillus viridinutans* CBS 12754 (100), *P. notatum* Tü 136 (30)	[38]
**38**	*Arthrobacter aurescens* ATCC 13344 (3), *B. subtillis* ATCC 6051 (10), *S. aureus* ATCC 11632 (10), *E. coli* K12 (10), *P. fluorescens* ATCC 13525 (0.1), *C. albicans* ATCC 10231 (10), *S. cerevisiae* Tü 125 (10), *A. viridinutans* CBS 12754 (10), *P. variotii* 137 (30), *P. notatum* Tü 136 (3)	[38]
**39**	*P. fluorescens* ATCC 13525 (3), *A. viridinutans* CBS 12754 (100), *P. notatum* Tü 136 (30)	[38]
**40**	*C. albicans* (25), *A. niger* (6.25), *P. chrysogenum* (7.81), *B. subtillis* (50), *S. aureus* (50), *M. luteus* (6.25), *M. canis* (1.95), *S. pyogenes* (100)	[63,64]
**41**	*C. albicans* (7), *A. niger* (3.13), *P. chrysogenum* (6.25), *B. subtillis* (15.62), *S. aureus* (12.50), *M. luteus* (3.13), *M. canis* (0.98), *S. pyogenes* (25)	[63,64]
**42** **43**	*B. cinereal* Persoon (ID_50_, 5), *Botryosphaeria dothidea* (ID_50_, 1.25); Diameter of inhibition zone (mm): *C. neoformans* 14 (22), *S. cerevisiae* 2399 (15), *saccharomyces sake yeast* (Papulacadnin B resistant strain) (20), *C. albicans* duke (20), *C. albicans* duke (Amphotericin B resistant strain) (23), *C. albicans* 3 (18), *C. tropicalis* (20), *M. gypseum* (20), *T. mentagrophytes* (25), *A. niger* (12)	[80]
**44**	*S. aureus* FDA 209P (12.5), *B. subtillis* NIHJ PCI 219 (12.5), *C. miyabeanus* IFO 5277 (3.2), *A. mali* IFO 8984 (12.5), *B. fuckeliana* IFO 5363 (1.6), *C. lagenarium* IFO 7513 (1.6), *P. filamentosa* sp. Sasakill IFO 6258 (1.6), *P. oryzae* IFO 5994 (6.25), *A. oryzae* IFO 5239 (25), *T. mentagrophytes* IFO 6202 (6.25), *S. cerevisiae* IFO 0304 (1.6), *C. albicans* IFO 1594 (1.6)	[66]
**46**	*P. infestans* (100), *Cladosporium fluvum* (25), *B. cinereal* (25), *P. oryzae* (25), *Cercospora beticola* (100)	[67]
**49**	*S. aureus* FDA 209P (25), *C. albicans* Yu 1200 (25)	[51,69]
**50**	*C. albicans* Yu1200 (12.5), *A. fumigatus* IAM 2046(12.5), *A. fumigatus* (IAM 2046) (MCC, 50)	[58]
**51**	Four isolates of *S. aureus* (0.50~1.00)	[70]
**52**	Four isolates of *S. aureus* (0.67~1.00)	[70]
**53**	Four isolates of *S. aureus* (0.67~0.83)	[70]
**54**	Four isolates of *S. aureus* (0.50~0.83)	[70]
**55**	Four isolates of *S. aureus* (0.25~0.50)	[71]
**56**	Four isolates of *S. aureus* (0.25)	[71]
**57**	Four isolates of *S. aureus* (0.25)	[71]
**58**	*S. aureus* FDA 209P (12.5), *B. subtillis* PCI 219 (25), 2 isolates of *C. albicans* IAM 4888 (25~50), *S. cerevisiae* IAM 4020 (50), *A. fumigatus* IAM 2153 (12.5), *M. racemosus* (12.5), *P. variotii* IAM 5001(12.5), *S. schenckii* (12.5), *C. albicans* Yu 1200 (MCC, >200)	[69]
**59**	*S. aureus* FDA 209P (6.25), *B. subtillis* PCI 219 (12.5), *C. albicans* IAM 4888 (25), *S. cerevisiae* IAM 4020 (50), *A. fumigatus* IAM 2153 (25), *M. racemosus* (6.25), *P. variotii* IAM 5001 (12.5), *S. schenckii* (25)	[69]
**60**	*S. aureus* FDA 209P (12.5), *B. subtillis* PCI 219 (25), *C. albicans* IAM 4888(25), *S. cerevisiae* IAM 4020 (50), *A. fumigatus* IAM 2153 (50), *M. racemosus* (25), *P. variotii* IAM 5001 (12.5), *S. schenckii* (25)	[69]
**61**	*S. aureus* FDA 209P (6.25), *B. subtillis* PCI 219 (12.5), *C. albicans* IAM 4888 (25), *S. cerevisiae* IAM 4020 (50), *A. fumigatus* IAM 2153 (25), *M. racemosus* (12.5), *P. variotii* IAM 5001 (12.5), *S. schenckii* (25)	[69]
**62**	*S. aureus* FDA 209P (0.78), *B. subtillis* PCI 219 (1.56), 3 isolates of *C. albicans* (3.12~25), *S. cerevisiae* IAM 4020 (6.25), *A. fumigatus* IAM 2153 (3.12), *M. racemosus* (3.12), *P. variotii* IAM 5001 (1.56), *S. schenckii* (3.12)	[69]
**63**	*T. mentagrophytes* (15.6), *T. rubrum* (15.6), *M. canis* (7.81), *C. albicans* (6.25~31.2), *A. niger* (15.6), *A. fumigatus* IAM 2046 (6.25), 3 isolates of *S. aureus* 6511 (12.5), *S. pyogenes* 308 (6.25), *S. pyogenes* 77 A (6.25), *S. faecium* D (50), *A. fumigatus* (MCC, 12.5)	[21,58,60,53]

**Table 4:** The acute toxicities of some guanidine-containing polyhydroxyl macrolides.

Compounds	Organisms	Test Type	Administration	Dose (mg/kg)	References
Copiamycin	mouse	LD_50_	Intraperitoneal	24.8	[76]
	mouse	LD_50_	Subcutaneous	61.5	[76]
Neocopiamycins A and B	mouse	LD_0_	Intraperitoneal	>1000	[50]
	mouse	LD_0_	Intravenous	>30 and >25	[50]
	mouse	LD_0_	Oral	>1000	[50]
Azalomycin F	mouse	LD_50_	Intraperitoneal	18 or 26	[87,88]
	mouse	LD_50_	Intravenous	12.5	[73]
	mouse	LD_50_	Oral	580	[73]
	mouse	LD_50_	Subcutaneous	162	[73]
Azalomycin F ^a^	mouse	LD_50_	Intraperitoneal	97.9	[25]
Guanidylfungin A	mouse	LD_50_	Intraperitoneal	12.5	[20]
Malolactomycin A	mouse	LD_50_	Intraperitoneal	6.7	[66]
Malolactomycin C	mouse	LD_0_	Intraperitoneal	>30	[67]
Malolactomycin D	mouse	LD_0_	Intraperitoneal	>30	[67]
RS-22 ^b^	mouse	LD_50_	Intravenous	25	[38]

^a^: a mixture of twelve azalomycin F analogs was used in the determination of LD_50_. ^b^: a mixture of RS-22 A, B, and C was used in the determination of LD_50_.
